# Phishing Website Detection Based on Deep Convolutional Neural Network and Random Forest Ensemble Learning

**DOI:** 10.3390/s21248281

**Published:** 2021-12-10

**Authors:** Rundong Yang, Kangfeng Zheng, Bin Wu, Chunhua Wu, Xiujuan Wang

**Affiliations:** 1 School of Cyberspace Security, Beijing University of Posts and Telecommunications, Beijing 100876, China; rundyang@bupt.edu.cn (R.Y.); binwu@bupt.edu.cn (B.W.); wuchunhua@bupt.edu.cn (C.W.); 2 School of Computer Science, Beijing University of Technology, Beijing 100124, China; xjwang@bjut.edu.cn

**Keywords:** URL, phishing detection, deep learning, random forest, ensemble learning

## Abstract

Phishing has become one of the biggest and most effective cyber threats, causing hundreds of millions of dollars in losses and millions of data breaches every year. Currently, anti-phishing techniques require experts to extract phishing sites features and use third-party services to detect phishing sites. These techniques have some limitations, one of which is that extracting phishing features requires expertise and is time-consuming. Second, the use of third-party services delays the detection of phishing sites. Hence, this paper proposes an integrated phishing website detection method based on convolutional neural networks (CNN) and random forest (RF). The method can predict the legitimacy of URLs without accessing the web content or using third-party services. The proposed technique uses character embedding techniques to convert URLs into fixed-size matrices, extract features at different levels using CNN models, classify multi-level features using multiple RF classifiers, and, finally, output prediction results using a winner-take-all approach. On our dataset, a 99.35% accuracy rate was achieved using the proposed model. An accuracy rate of 99.26% was achieved on the benchmark data, much higher than that of the existing extreme model.

## 1. Introduction

Phishing attacks have become a significant concern owing to an increase in their numbers. It is one of the most widely used, effective, and destructive attacks, in which attackers try to trick users into revealing sensitive personal information, such as their passwords and credit card information. A typical phishing attack technique involves using a phishing website, where the attacker lures users to access fake websites by imitating the names and appearances of legitimate websites, such as eBay, Facebook, and Amazon.

**Figure 1:**
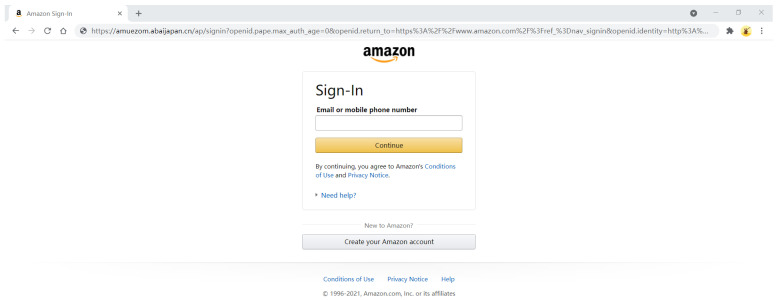
Example of phishing website.

As shown in Figure 1, it is difficult for the average person to distinguish phishing websites from normal websites because phishing websites appear similar to the websites they imitate. In many cases, users do not check the entire website URL, and, once they visit a phishing website, the attacker can access sensitive and personal information.

With the growth in the field of e-commerce, phishing attack and cybercrimes are rapidly growing. Attackers use websites, emails, and malware to conduct phishing attacks. According to the Anti-Phishing Working Group (APWG) Q4 2020 report, in 2020, there was an average of 225,759 phishing attacks per month, an increase of 220% compared to 2016 [1]. The country most affected by phishing sites is China, with 47.9% of machines infected. Phishing has become one of the biggest threats in cybersecurity. According to the FBI Internet Crime Center data records, the economic loss due to phishing crimes can reach $3.5 billion in 2019 [2].

**Figure 2:**
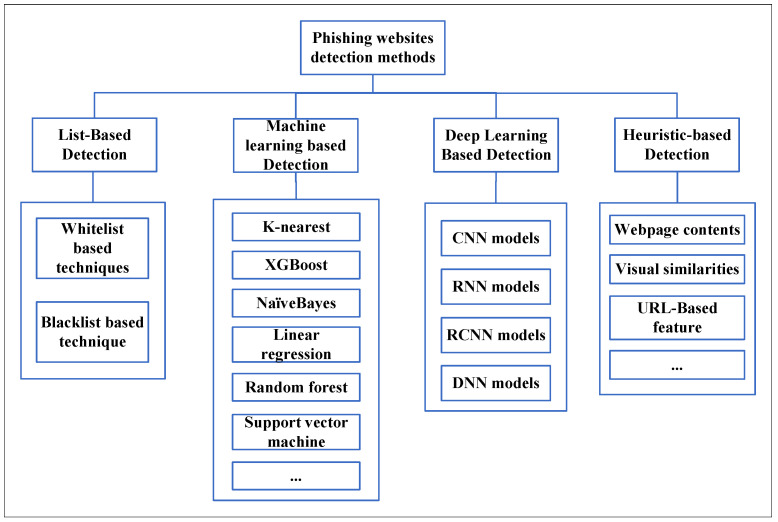
Category of phishing detection techniques.

Phishing crimes are usually underreported. New phishing detection techniques have been developed to mitigate phishing attacks. A detailed review of the methodologies of various anti-phishing papers is given by Mohammad et al. [3]. Phishing website detection techniques are categorized into four types, whitelist/blacklist-based techniques, deep learning-based detection, machine learning-based detection, and heuristic-based detection techniques, as described in Figure 2.

The black and whitelist phishing website detection technology has a high detection speed. However, the major disadvantage is that the detection rate relies on the number of websites included in the black and whitelist; moreover, it cannot resist zero-day attacks [4,5,6,7]. Heuristic detection technology was later proposed to detect phishing websites by extracting features of multiple web pages and third-party services, among which third-party service features include website ranking, network traffic detection, and WHOIS information, to resolve issues with blacklist techniques [8,9,10,11]. The detection is time consuming, and the accuracy is not exceptional owing to the difficulties associated with third-party service feature extraction. Machine learning-based phishing website detection uses machine learning methods to detect manually extracted phishing website URL features. The efficiency of detection can be improved using this method. This semi-automatic method requires experts to extract URL features manually, build a training set for phishing website detection, and, finally, use supervised learning methods to detect a phishing website. It requires updating URL features owing to the frequent changes in the URL structures, requiring professional operation and high maintenance costs [12,13,14,15,16,17,18,19,20].

Deep learning can effectively overcome the problem of manual feature extraction. Convolutional neural networks (CNN) [21] are one of the most widely used deep learning models showing good performance in feature learning and phishing website detection. Generally, the last convolutional layer in the CNN network is used as the classification feature, which is transmitted to the classifier for classification. The last layer features contain global and high-latitude features for effective category classification; however, they are not very effective for URL-based phishing website classification. Several studies have used CNN models with multi-level and multi-scale feature aggregation, effective in phishing website detection and other applicationss [22,23,24,25,26]. These models use multi-scale features aggregated into a total feature, which is then used as a fully connected layer input for classification. However, they use some of the multilayer features. In this paper, we design a URL-based character-level CNN embedding model that makes full use of multilayer features.

The proposed model takes URL strings as input, identifying each character in the training set based on a prescribed character vocabulary, and using character embedding to represent each character as a fixed-length vector. This matrix is input into the CNN model. It then automatically extracts representative multi-level features from these vectors using the proposed improved CNN model. Finally, it classifies phishing websites separately using multiple RFs for different features extracted by CNN. It outputs phishing website detection results by aggregating the outputs of multiple classifiers using a winner-take-all strategy.

The main advantages of the proposed method are listed as follows:

1. Strong generalization ability: The proposed method has strong generalization ability. The multi-level features used by the proposed method obtain better generalization ability and check-side accuracy. The low-level features in the hidden layer are common and similar for different but related distributed datasets or tasks; these are combined with the low-latitude features in the hidden layer.
2. Third-party service independence: The proposed method relies only on website URL features for detection, without extracting third-party features, such as page rank, search engine index, web traffic measurement, and domain age, which can improve the efficiency of detection and reduce the detection time.
3. Independence of cybersecurity experts: Reduced required expert function engineering, the deep neural network CNN model proposed in this paper can automatically extract URL features without the need for experts.
4. Language-independent: The approach proposed in this paper is effective for the detection of websites with content in various languages using character-level features.

The main contributions of this paper are as follows.

1. This paper proposes a phishing website detection technique based on integrated learning and deep learning with fast and accurate detection of phishing websites using only URL features.
2. We built a real dataset by crawling 22,491 phishing URLs from phishtank and 24,719 legitimate URLs from Alex and conducted experiments on the dataset.
3. The phishing website detection process based on ensemble learning and deep learning is described, and the constructed dataset is extensively experimented. The results of the experiments indicate that our proposed method shows good performance in terms of accuracy and false positive rate.

The remainder of the paper is organized as follows: Section 2 introduces some problems related to phishing website detection, Section 3 introduces character embedding, CNN, RF, and the phishing website detection method proposed in this paper, Section 4 analyzes the experimental results of the proposed method, and Section 5 provides the conclusion and future scope of this work.

## 2. Literature Review

Although attacks use different techniques to create phishing websites to deceive users, most have similarly designed phishing website features. Therefore, researchers have conducted extensive anti-phishing research using phishing website features. Current methods for phishing detection include black and whitelists, heuristics, visual similarity, and machine learning, among which heuristics and machine learning are more widely used. The following is an introduction to the aforementioned phishing detection techniques.

1. Black and whitelist

To prevent phishing attack threats, many anti-phishing methods have been proposed. Blacklisting methods are the most straightforward ways to prevent phishing attacks and are widely used in the industry. Google Safe Browsing uses a blacklist-based phishing detection method to check if the URL of the matching website exists in the blacklist. If it does, it is considered a phishing website; otherwise, it is a legitimate website. Jain and Gupta [27] proposed an automatic update whitelist technique to prevent phishing attacks in 2016. This method uses the hyperlink function to check the legitimacy of web pages; it extracts the hyperlinks from the source code of the web pages when accessed, and applies them to a phishing detection algorithm. This method can effectively detect various types of phishing attacks. Lung-Hao and Kuei-Ching et al. proposed a framework to automatically update the blacklist of phishing websites, PhishTrack, in 2014 [28]. This framework explores existing blacklists to discover suspicious URLs. PhishTrack includes redirect and form tracking components to update phishing blacklists, and it proactively discovers phishing URLs as early as possible. This proactive phishing update approach effectively improves blacklist coverage and complements existing anti-phishing techniques to provide safe web surfing.

Black and whitelist-based phishing detection has high accuracy and can prevent phishing attacks, to some extent. It has low system overhead for fast client access only if they are included in the blacklist of phishing websites. However, phishing websites can be created at any time and place, and their average life span may be a few hours; the black and white list-based phishing detection approach in itself has low efficiency in prevention from these types of attacks [29]. Other technical means are needed to detect websites not detected by this method.

2. Heuristic

Zhang et al. proposed CANTINA in 2007, which is a content-based phishing detection. The authors used the TF-DF method to identify phishing websites, where the first five terms based on TF-DF are sent to the search unit for comparison with the results obtained by the search unit using linkable links [19]. This model applies to web pages consisting of text content. However, the detection accuracy of this model decreases when the text on the web page is replaced with an image. Heuristics, which provide us with the rules in the if-then form, are applied by Reference [30] for association classification mining.

Rao and Ali proposed a heuristic approach to phishing detection in 2015 called PhishShield, a desktop application that focuses on phishing detection using the URL and website content of phishing websites [9]. The features extracted by PhishShield are mainly null-valued footer links, zero links in the HTML body, copyrighted content, title content, and website logos. Compared with the black and whitelist method, the PhishShield is faster, more accurate, and has a more comprehensive detection range for detecting phishing websites. However, the detection efficiency decreases when the attacker understands the heuristic technique and can effectively bypass the heuristic filter.

3. Visual Similarity

To convince users of the legitimacy of a website, phishing attackers build websites with high similarity to the content of their target pages, which is mainly manifested in the logo, Favicon, CSS architecture, page layout, and overall visuals of the web page. The visual similarity-based approach compares the visual content of suspect websites and the visual content of trusted domains. It determines if it is a phishing website by comparing similarity results.

Zhang et al. proposed a visual similarity-based phishing website detection method, which uses the spatial features of web pages as a basis for detection [31]. Mao et al. saved web pages in standard image forms, segmented the images, and used the EMD algorithm to compare the target web pages to analyze the visual similarity between target and known web pages for determining phishing websites [32].

Yun Lin et al. [33], using visual similarity technique, designed a hybrid deep learning system that does not require training on fishing samples, which is better than previous visual similarity methods.

Visual similarity-based methods can detect phishing sites to some extent. However, most web content is not constant, and, once the features of a web page change, the method results in a detection error. Compared with the aforementioned methods, this method cannot correctly handle the changing phishing web pages and is slower. The change in the user interface of these web pages can result in different analyses causing false positive and false negative results.

4. Machine Learning

Machine learning detection techniques overcome the shortcomings of the aforementioned methods. Machine learning algorithms are used to classify and identify suspicious phishing feature values to simulate manual analysis to identify illegal websites automatically. It is necessary to extract phishing website feature characteristics and training models to improve machine learning accuracy. In 2011, Xiang et al. proposed CANTINA+, a model for detecting phishing websites, based on CANTINA with eight new features [15]. Zouina et al. proposed a novel lightweight phishing detection based entirely on URLs in 2017 [34]. Toolan proposed a method to select the best features using information gain. Forty phishing website detection features were extracted, and the best features were then selected from these using the information gain method for phishing website detection [35]. OFS-NN uses the FFV index, with which the importance of each feature for detection can be evaluated; finally, the best feature is selected for phishing site detection [36]. A phishing website detection system was implemented by Mohammad, Thabtah, and McCluskey [37]. The system uses an adaptive self-constructing neural network for classification.

## 3. Proposed Method

**Figure 3:**
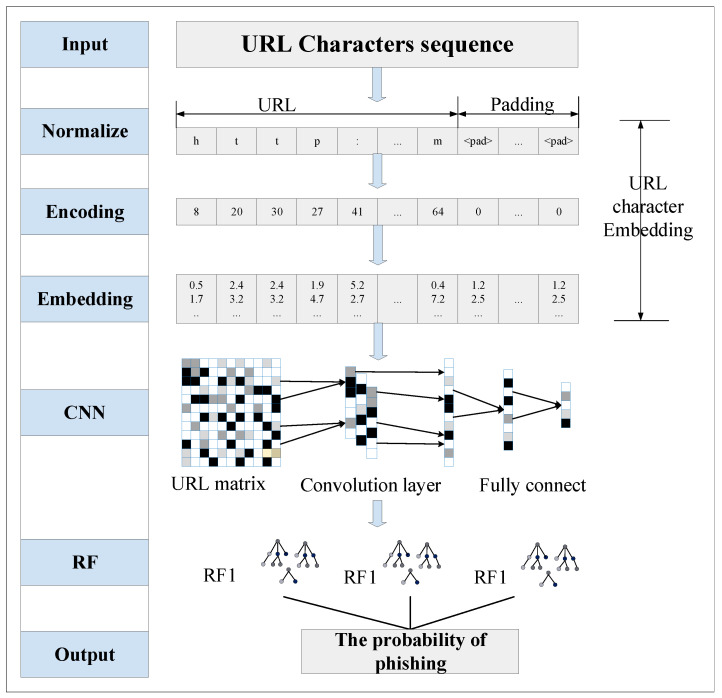
Framework of the proposed method.

This section presents a phishing website detection method based on character embedding, CNN, and RFs. The overall structure of the proposed method is shown in Figure 3.

The phishing website detection method proposed in this paper consists of three main components. First, URL data is transformed into a character vector using the character embedding method. The converted URLs have the same data structure, which is beneficial for the detection of phishing websites. Second, an improved CNN network is designed, and the model is trained using the transformed URL data. After the model is trained, the URL features are extracted to obtain the features of different layers in the CNN network. Third, the features extracted from different network layers are classified in random forests separately. The classifier with the best classification result is used as the final classifier to classify the website.

### 3.1. URL Character Embedding

This paper uses the character embedding method [38] to embed URLs by expanding the characters. The main reasons for using character embedding instead of word embedding are as follows. The total number of characters is fixed; hence, there is no possibility of failing to extract features owing to the presence of new words. Phishing website URLs usually use nonsensical words; and, as URLs are processed on characters, they are not restricted by language and can be used on any language.

Usually, the URLs of phishing websites imitate the URLs of normal websites, and attackers confuse users by making minor changes to the URLs. For example, by using similar characters, goole.com is changed to gooIe.com, and the character “l” is replaced with “I.” More information can be included at the character level, and character-level embedding can better detect small changes in URLs, improving the detection performance of phishing sites.

The creation of encoding for the alphabet is the first step for the implementation of URL character embedding. The alphabet used in this paper has 96 parameters, including 26 lowercase letters, 26 uppercase letters, 10 numbers, 32 other characters, and the ”unrecognizable character <UNK>” and “fill character <PAD>”, as shown in Table 1. In the character count, if the number of characters counted is less than 50, the character is considered unrecognizable and is replaced using <UNK>. URLs have different lengths. Hence, we set a uniform length of L = 200; if the URL character length exceeds 200, only the first 200 characters are considered, whereas, if the URL character count is less than 200, the characters are filled till 200 with <PAD>.

In our work, each URL character is embedded in a 32-dimensional vector using the character embedding method. The embedded vector is randomly initialized for learning during the training of the model. To facilitate the manipulation of the data, the data is stored using the matrix $EM\in R^{L_{1}*K}$.

$$u\mapsto x\in R^{L_{1}\times k},$$

where k=32, $L_{1}=200$.

### 3.2. Designing an Improved CNN

An improved CNN network is designed and trained based on the transformed URL matrix using the character embedding method. The CNN network is trained using the URL training set, and multiple model parameters are continuously updated by the back-propagation method. After the model training is completed, multilayer URL features are extracted from the CNN network.

**Figure 4:**
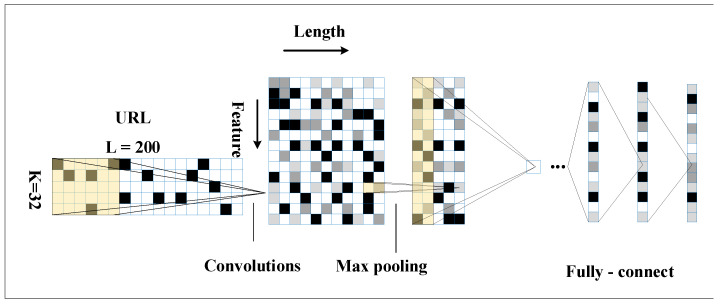
Improved CNN.

Figure 4 introduces the architecture of the improved CNN network. It has seven layers. The first to seventh layers are the input, convolutional, pooling, linear 1, linear 2, linear 6, and output layers.

The traditional CNN network structure was adjusted to ensure that the input URL data contains enough URL information. The URL length was set to 200, and the input layer was convolved using 256 convolutional kernels of 5 × 32. The data obtained was of size 256 × 196 × 32 and activated using the ReLU function. It was then pooled using size 256 × 32 with a linear layer of 512. The activation was carried out using ReLU with a linear layer 2 of size 256. The activation was further performed using ReLU and a linear layer 3 of size 128. Finally, the output of the result was obtained using the SoftMax classifier.

In the final linear layer L3, the feature mapping is classified after SoftMax to give its probability value determining whether it is a phishing website or not. The formula is calculated as follows.

(1) $$p\left(y^{\left(i\right)}=j|x^{\left(i\right)};\theta \right)=\frac{\exp\left(\theta_{j}^{T}x^{\left(i\right)}\right)}{\underset{k}{\overset{l=1}{\sum^{}}}\;\exp\left(\theta_{l}^{T}x^{\left(i\right)}\right)},$$

(2) $$y=argmax_{j}p\left(y^{\left(i\right)}=j|x^{\left(i\right)};\theta \right),$$

where i=1,2,3…,n,i denotes the number of training data, j=1,2,3…,n,j denotes the dimensionality of the output layer, and the output layer is set as the number of website types in this paper. Furthermore, θ denotes the classification parameter of the SoftMax classifier, and the loss function of the SoftMax classifier is defined as

(3) $$J\left(\theta \right)=-\frac{1}{n}\left[\underset{n}{\overset{i=1}{\sum^{}}}\;\underset{k}{\overset{j=1}{\sum^{}}}\;I\left\{y^{\left(i\right)}=j\right\}\right]\log p\left(y^{\left(i\right)}=j|x^{\left(i\right)};\theta \right),$$

where J(θ) is the cross-entropy loss function. The gradient descent method is used to solve for the minimum of the J(θ) function and optimize the CNN network parameters.

The general procedure of extracting multilayer features based on the proposed CNN is given in Algorithm 1.
**Algorithm 1** Extract Multiplyer Features (EMF-CNN)**Input**: The training dataset $S_{train}$, The testing dataset $S_{test}$, $s_{i}\in S_{\mathrm{train}\;}$, $s_{i}^{\prime }\in S_{\mathrm{test}\;}$.**Output**: Multiplyer features F1, F2, F3.    1:  *t* = size of sliding step, β = threshold value of loss function L(x,y), *T* = num of   sliding-window, W=weight, $X\in R^{m*n}$2:  $S=S_{\mathrm{train}\;}\cup S_{\mathrm{test}\;}$, l=|S|, X=∅, M=⌈T/p⌉3:  **For**
*i* in *l* do4:       $s_{i}\in S$5:       $m_{i}=Characterembedding\left(s_{i}\right)$6:       $X=X\cup m_{i}$7:  **end for**8:  $\left.X=\vec{\left(x_{1}\right.},\vec{x_{2}},\ldots ,\vec{x_{n}}\right.)$9:  **For**
*j* in *B* do10:       **For** *i* in *D* do           $h_{i}^{j}=\sigma \left(W_{j}\cdot \left(\vec{x_{l}},\vec{x_{l+1}},\ldots ,\vec{x_{l+t-1}}\right)+b_{j}\right)$11:       **end for**12:  **end for**13:  **For**
*n* in *B* do14:       **For** *t* in *M* do15:          $p_{n}^{j}=\mathrm{Max}\left(h_{(n-1)p}^{j},h_{(n-1)p+1}^{j},\ldots ,h_{np-1}^{j}\right)$16:       **end for**17:  **end for**18:  $H_{p}={\left(p^{1},p^{2},\ldots ,p^{j},\ldots ,\vec{p^{s}}\right)}^{T},p^{j}\in R^{N\times 1}$19:  $C^{\prime \prime }=\mathrm{softmax}\left(h_{p}\right)$20:  while $L\left(C^{\prime \prime },C\right)>\beta$21:       $W=Train(s_{i},C_{i})$22:  end while23:  $\left(F1,F2,F3\right)\left.=\vec{\left(p^{2}\right.},\vec{p^{3}},\vec{p^{5}})\right.=Train\left(s_{i},\;W\right)\cup \mathrm{Test}\left(\left(s_{i}\cup s_{i}^{\prime }\right),W\right)$24:  **return**
 (F1,F2,F3)

### 3.3. Ensemble Classification

**Figure 5:**
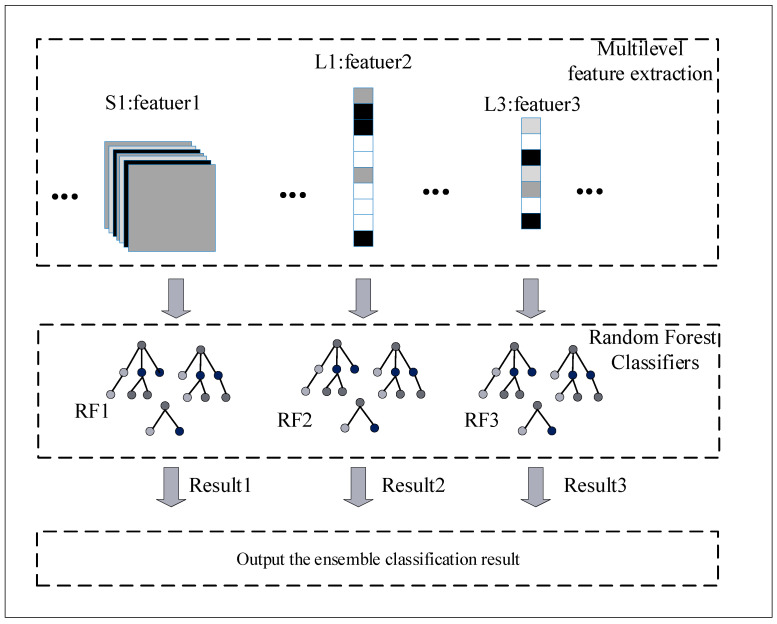
Ensemble classifiers.

The classification of phishing websites can be achieved using multi-level features to improve the accuracy and generalization ability of the classification algorithm. In this paper, multi-level URL features are extracted from the improved CNN network, as shown in Figure 5, and URL features are extracted using the pooling layer, L1 layer, and L3 layer. The aforementioned features are classified using an RF classifier, respectively. Each RF classification contains 100 decision trees with a maximum depth of 5 in the child nodes, where the CNN network is used to extract URL multi-level features and RF to classify multi-level features.

In order to extract more comprehensive URL feature information, high latitude features, mid-latitude features, and low latitude features are extracted separately. In addition, ensemble learning has a great impact on the performance improvement of the model and is widely used. So, three RFs are used to classify these features.

For different RF classifiers, features are extracted from different CNN network layers and used as training data for the RF classifiers. The results of each classifier are output after the training of the three RF classifiers is completed. The best RF classification result is used as the final classification result of phishing websites. Using this classification strategy of combining multiple classifiers can improve the accuracy and increase the generalization ability of the phishing website detection model. Using the max voting strategy, the output results are consistent between all ensemble classifiers and the base classifier. The best classification results are obtained in different layers.

The general procedure of the phishing website classification method based on the combination of improved CNN network and multiple RFs is given in Algorithm 2.
**Algorithm 2** General procedure of proposed method**Input**: Set of URLs $S=\left\{s_{1},s_{2},\ldots ,s_{n}\right\}$**Output**:The probality of phishing P(S)    1:  M=|S|, H1=∅, H2=∅, H3=∅2:  **For**
*j* in *M* do3:       F1=∅, F2=∅, F3=∅4:       $F1,F2,F3=EMF-CNN(s_{i})$5:       H1=H1∪F1, H2=H2∪F2, H3=H3∪F36:  **end for**7:  P(U)=max(RF(H1),RF(H2),RF(H3))8:  **return**
 P(U)

## 4. Experimentation and Result Analysis

This section will introduce the details of the model and analyze the experimental results. To evaluate the effectiveness of the proposed method in phishing detection, two phishing datasets were analyzed and studied separately. All experiments were conducted using PyCharm on a laptop equipped with an Intel 8-core 2.3 Ghz processor, GTX3060 graphics card, 16 GB RAM, and 512 GB hard disk.

### 4.1. Dataset

To evaluate the classification accuracy of phishing websites by the model proposed in this paper, two datasets, D1 and D2, are used. Dataset D1 contains 47,210 URLs, with 24,719 URLs for legitimate websites and 22,491 URLs for phishing websites. Legitimate websites were collected from the ALEXA (https://www.alexa.com/) and phishing websites from PhishTank (https://phishtank.org/), which mainly collected the websites that have been verified from January 2021 to June 2021. D2 is the dataset used in the literature [12], containing 83,857 URLs, of which 43,189 are legitimate websites from Yandex and 40,668 are phishing websites from PhishTank. The distribution of data is shown in Table 2.

**Figure 6:**
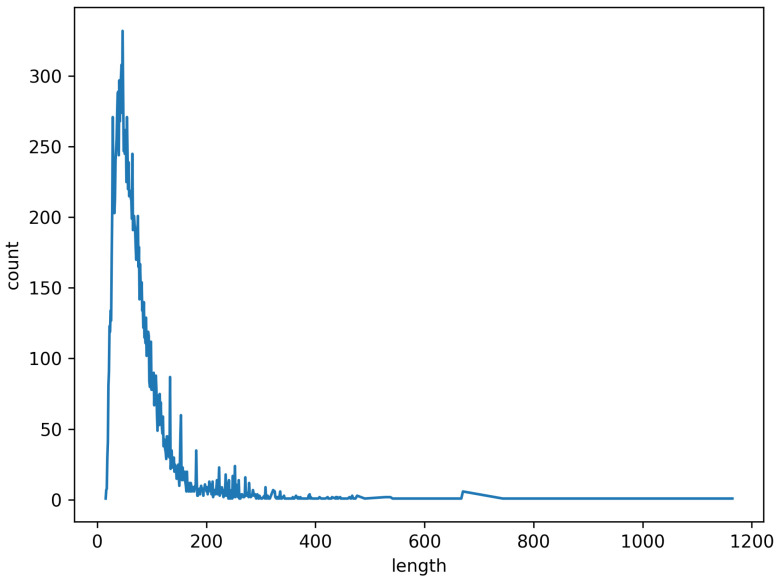
URL length of D1.

Figure 6 shows the length distribution of URLs in dataset D1. The figure shows that the length of URLs is mostly concentrated in the range 0–100, and the longest URL in this dataset is 1200. To standardize the URL length, the URL length of 200 is selected in this paper.

We use statistical indicators to evaluate our model effectively, including sensitivity, recall, accuracy, precision, F1_core, and AUC, calculated as follows.

(4) Accuracy=(TP+TN)/(TP+TN+FP+FN),

(5) Precision=(TP)/(TP+FP),

(6) Recall=(TP)/(FP+FN),

(7) F_Measure=2∗precision∗recall/(precision+recall),

where *TP* is the number of URLs marked as phishing in the data that are classified as phishing URLs, *TN* is the number of URLs marked as legitimate in the dataset that are classified as legitimate URLs, *FP* is the number of URLs marked as legitimate in the dataset that are classified as phishing URLs, and *FN* is the number of URLs marked as phishing in the dataset that are classified as legitimate URLs.

AUC and ROC are important metrics for evaluating the binary classification model.The horizontal coordinate in the ROC curve is FPR, which indicates the probability that URLs marked as legitimate in the dataset are classified as phishing URLs, and the vertical coordinate is TPR, which indicates the probability that URLs marked as phishing URLs in the dataset are classified as phishing URLs. It is defined as shown below.

(8) FPR=(FP)/(FP+TN),

(9) TPR=(TP)/(FP+TN).

### 4.2. Experimental Setup

The padding method normalized the original URL data, where the URL length is uniformly set to 200. All URLs are converted into a 32 × 200 size vector by the character embedding method. In this paper, the detailed structure of the CNN structure in the proposed improved CNN network is shown in Table 3; moreover, it can be seen that the CNN structure has the best performance in the configuration. The C1 layer (256@192 × 1) indicates that there are 256 @192 × 1 feature maps, where C1(256@5 × 32) indicates that the C1 layer is obtained by computing 256 convolutional kernels of size 5 × 32. S2 (2 × 1) indicates that it is obtained in the C1 layer using a pooling operation of size 2 × 1. The model parameters of the CNN are optimized using a heuristic optimization method for the setting of the parameters. The initial learning rate is set to 0.01, and the learning step is set to 0.05. To have the best performance of the proposed model, the batch size is set to 64, and the epoch is set to 200, according to the size of the sample.

### 4.3. Evaluation on D1 with Different CNN Models

In this experiment, different CNN models are evaluated on D1 using CNN1, the proposed convolutional neural network (CNN). The structure of the CNN1 model is shown in Table 4. The primary goal of this experiment is to reveal the best CNN model and parameters suitable for URL feature extraction.

**Figure 7:**
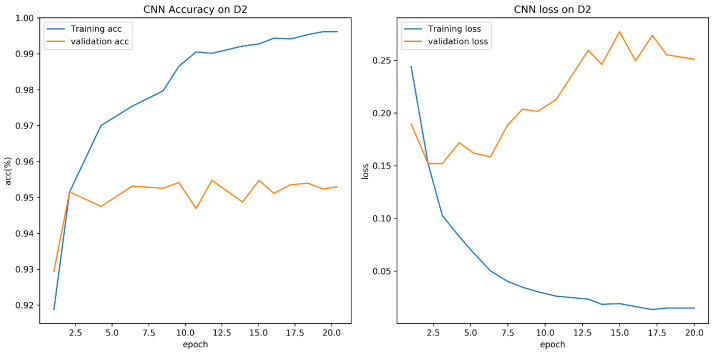
Evaluation on D1 with CNN.

**Figure 8:**
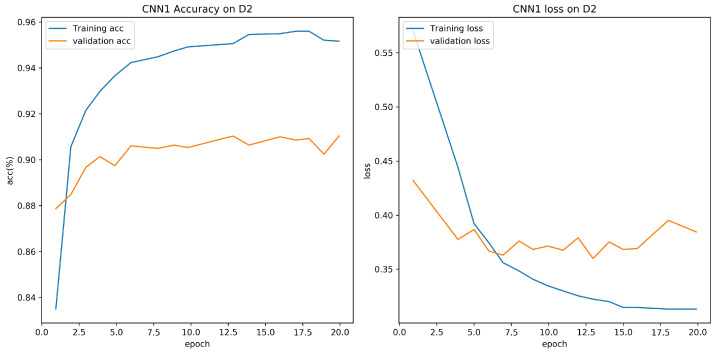
Evaluation on D1 with CNN1.

Experiment results are shown in Table 5. From the experiment result, it is clear that accuracy for CNN model proposed in this paper is 95.73% on D1, which is higher than CNN1 model. The training batch size is set to 64, and the number of epoch is set to 20. Figure 7 and Figure 8 show the validation loss and accuracy of the CNN and CNN1 training and testing data, respectively.

### 4.4. Evaluation on D1, D2 with RNN and CNN

**Figure 9:**
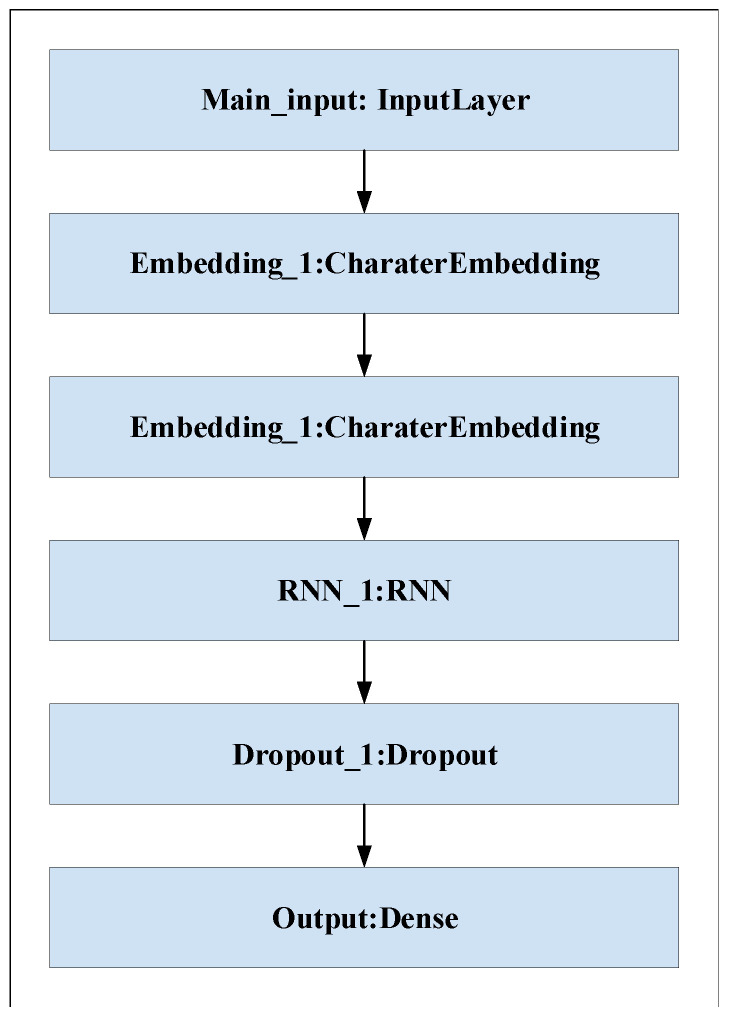
The structure of RNN.

**Figure 10:**
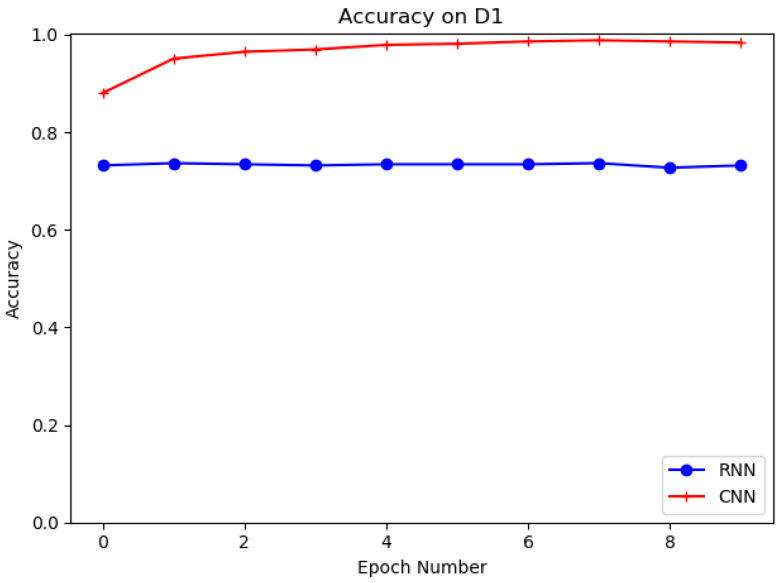
Evaluation on D1 with CNN, RNN.

**Figure 11:**
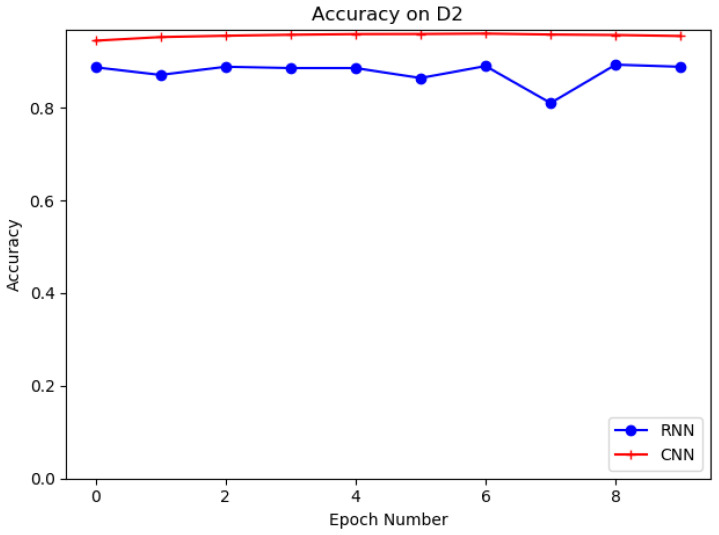
Evaluation on D2 with CNN, RNN.

Different deep learning models, such as recurrent neural network (RNN) and proposed convolutional neural network (CNN), are evaluated in this experiment on datasets D1 and D2. The structure of RNN is shown in Figure 9. The purpose of the experiments is to select the best deep learning model for URL feature extraction. Table 6 shows the results of CNN and RNN models on D1 and D2. It is clear that the CNN model has the higher accuracy and the better detection performance on both datasets. It can be seen that the CNN model has 95.73% and 94.45% accuracy for D1 and D2, which is higher than the RNN model for D1 and D2 with 72.3% and 88.75% accuracy. In training, we set the training batch size to 64 and the number of epochs to 10. Figure 10 and Figure 11 show the accuracy of CNN and RNN for datasets D1 and D2, respectively.

### 4.5. Evaluation on D1 with Different Classifier

From previous experiments, CNN models outperform other deep learning models in classifying phishing websites. Therefore, in this experiment, different classifiers are evaluated using CNN deep learning models. In this experiment, we compare different classifiers, such as plain Bayesian (MNB), logistic regression (LR), Xgboost (XGB), and random forest (RF). We then extract multilayer features, S2, L3, L4 layer features using the CNN model in this paper on D1 dataset. Subsequently, we classify the extracted features with different classifiers and calculate the final classification using the winner-take-all method results. The main objective of this experiment is to expose the best-integrated classifier suitable for URL features. The experimental results are shown in Table 7, according to which the RF classifier has good accuracy, precision, F-Score, and AUC. The RF classifier is an ensemble classifier that turns weak learners into strong ones for various features with different dimensions. It is used in D1 with 99.35% accuracy, 99.34% F-Score, and 99.34% of AUC outperformed other classifiers.

**Figure 12:**
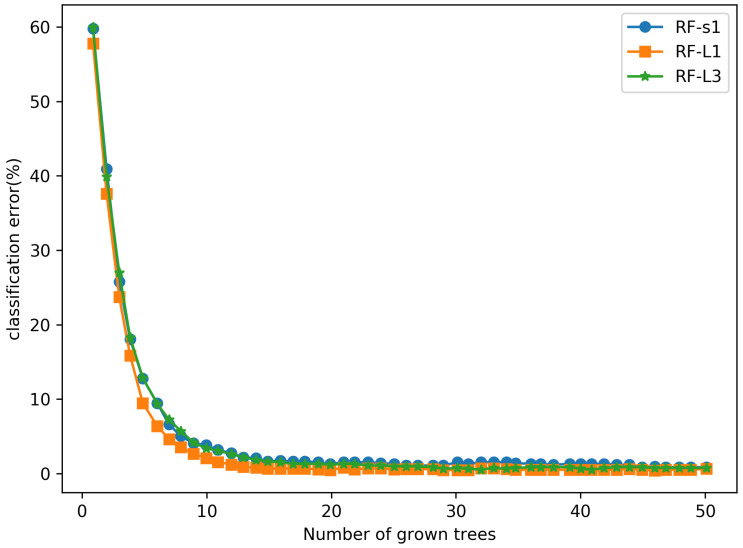
The training error curves of 3 RF classifiers.

The extracted URL features are fed into the three RF classifiers separately, and the training error curves are shown in Figure 12. It is important to note that the training errors of all three RF classifiers are close to zero, which indicates further that the feature mapping maps in the implicit layer also contain the significant information that contributes to the phishing websites detection results.

### 4.6. Comparison of Proposed Model with Existing Baselines

In this experiment, the aims are to compare the phishing website classification model proposed in this paper with the models proposed by Sahingoz et al. [12], Rao et al. [13], and Le et al. [14], to evaluate the effectiveness of our proposed model. Le et al.’s algorithm was implemented on dataset D2. We applied our model on dataset D2 for better comparison with Raoand Sahingoz et al. These models were selected for comparison in this experiment because of the similarity of the phishing site detection techniques they use. They all use URLs for phishing site detection without accessing the website’s content.

In their paper, Rao et al. applied their proposed method to dataset D2 and achieved 98.25% accuracy, 98.23% F1-score, and 98.04% precision. Since the existing literature does not have an implementation of Le et al.’s algorithm on dataset D2, in order to ensure the fairness of the comparison, we applied Le et al.’s algorithm to dataset D2 and achieved 95.49% accuracy, 95.27% F1 scores, and 96.78% precision. Finally, we applied our method to dataset D2. Our method performed better than other methods, obtaining an accuracy of 99.26%, precision of 99.19%, F1 score of 99.23%, and sensitivity of 99.28%, which shows that our phishing website detection method outperforms existing methods. The results of our proposed model compared with the existing baseline model are shown in Table 8.

According to the results, it can be seen that the accuracy of phishing website detection by traditional machine learning-based methods on dataset D2 is significantly lower than that of the proposed method, which is due to the shallow architecture of traditional machine learning-based methods that cannot explore the complex relationship between URLs and phishing. In addition, the performance of these phishing site detection methods relies heavily on manual feature extraction. However, manually extracted phishing website features have poor ability to represent the raw data. The method also achieves better results compared to other deep learning-based methods, further demonstrating the superiority of the method. The superior performance of the method is mainly attributed to the strong automatic feature learning capability of the proposed CNN model and the generalization capability of the ensemble classifier.

## 5. Conclusions and Future Work

In this paper, we proposed a multi-level feature phishing website classification method based on character embedding CNN and RF. The main features of this model is as follows.

(1) Character embedding of URLs is performed to convert URLs into normalized matrices, containing much important phishing website classification information in the URL characters. This information helps classify phishing websites. URLs are transformed into uniform signals by the character embedding technique, more suitable for CNN networks’ input.
(2) Automatic phishing web feature extractor using CNN. The CNN model is pre-trained using the converted URL data to optimize and improve the CNN model parameters. The pre-trained model can extract multi-level features from the URL data. The extracted multi-level features contain sensitive information that can classify phishing websites and provide knowledge for phishing website classification.
(3) Using multiple RF classifiers and a winner-take-all strategy improves the model’s accuracy and generalization. Extracting multi-level features for low latitude can be used to classify phishing websites. The RF classifier is trained using the extracted features of each layer, outputting the results of each RF, and, finally, choosing the one with the best results, improving the classification results.
(4) The proposed method in this paper is validated by the dataset from PhishTank and Alex. A 99.35% correct classification rate of phishing websites was obtained on the dataset. Experiments were conducted on the test set and training set, and the experimental results proved that the proposed method has good generalization ability and is useful in practical applications.

Although the proposed method in this paper has achieved some good results, there are still some shortcomings. The main disadvantage is that it takes longer to train. However, the trained model is better than the others in terms of accuracy of phishing website detection. Another disadvantage is that the model cannot determine whether the URL is active or not, so it is necessary to test whether the URL is active or not before detection to ensure the effectiveness of detection. In addition, some attackers use URLs that are not imitations of other websites, and such URLs will not be detected. The next step of our work aims to use new techniques to automatically extract other features for detecting phishing sites, such as web code features, web text features, and web icon features.

## Figures and Tables

**Table 1:** Unique characters.

Unique Characters
abcdefghijklmnopqrstuvwxyz
ABCDEFGHIJKLMNOPQRSTUVWXYZ
-:;.!?/[] ()@=+ # $ % & { } _ ^ - <>|\
0123456789
Unrecognizable
Padding

**Table 2:** URL Dataset used in our model.

Date Name	Legitimate URL	Source	Phishing URLS	Source	Total
DATA1	24,719	ALEXA	22,491	PhishTank	47,210
DATA2	43,189	Yandex	40,668	PhishTank	83,857

**Table 3:** The detailed structure of improved CNN model.

Layer Name	Configuration	Kernel/Pooling Size
Input	32 × 200	
C1	256@196 × 1	256@5 × 32
S2	256@1	256@196 × 1
L3	512	
L4	256	
L5	128	
FC6	128	
Output	2	

**Table 4:** The detailed structure of improved CNN1 model.

Layer Name	Configuration	Kernel/Pooling Size
Input	95 × 95	
C1	32@6 × 6	32@3 × 3
C2	6@3 × 3	6@2 × 2
C3	16@5 × 5	16@2 × 2
C4	120@5 × 5	
FC5	84	
Output	2	

**Table 5:** Evaluation on D1 with CNN and CNN1.

Sets	Model	Accuracy for DATA1 (%)	F1 (%)	Precision (%)	Recall (%)	AUC (%)
D1	CNN	95.73	95.53	95.96	95.11	95.37
	CNN1	90.34	89.96	90.87	88.06	90.23

**Table 6:** Evaluation on D1, D2 with RNN and CNN.

Sets	Model	Accuracy for DATA1 (%)	F1 (%)	Precision (%)	Recall (%)	AUC (%)
D1	CNN	95.73	95.53	95.96	95.11	95.37
	RNN	72.32	71.76	73.85	69.79	72.18
D2	CNN	94.45	94.30	94.85	93.37	94.21
	RNN	88.75	88.53	89.56	87.53	88.46

**Table 7:** Results of the classification models on D1.

Model	Feature	Accuracy for DATA1 (%)	F1 (%)	Precision (%)	Recall (%)	AUC (%)
MNB	CNN + MNB1	79.28	77.46	74.77	80.36	79.11
	CNN + MNB2	79.23	78.93	81.68	76.36	79.05
	CNN + MNB3	79.24	78.67	80.39	77.03	79.31
	CNN + MNB	79.28	77.46	74.77	80.36	79.11
LR	CNN + LR1	86.33	85.85	87.08	84.65	86.16
	CNN + LR2	86.29	86.01	88.48	83.67	86.21
	CNN + LR3	86.21	85.92	87.15	84.73	86.33
	CNN + LR	86.33	85.85	87.08	84.65	86.16
XGB	CNN + XGB1	89.27	88.68	88.28	89.08	89.26
	CNN + XGB2	89.31	89.29	88.90	87.92	89.30
	CNN + XGB3	89.12	88.31	86.29	90.42	89.32
	CNN + XGB	89.31	89.29	88.90	87.92	89.30
**RF**	CNN + RF1	99.25	99.25	99.15	99.11	99.24
	CNN + RF2	99.35	99.34	99.52	99.21	99.34
	CNN + RF3	99.27	99.26	99.14	99.06	99.27
	**CNN + RF**	**99.35**	**99.34**	**99.52**	**99.21**	**99.34**

**Table 8:** Comparison of the proposed model with existing baseline models on D2.

Metrics (%)	Sahingoz et al. [12]	Rao et al. [13]	Le et al. [14]	Our Method
Precision	97.00	98.04	96.78	**99.19**
Sensitivity	99.00	98.42	95.24	**99.28**
F-measure	98.00	98.23	95.27	**99.23**
Accuracy	97.98	98.25	95.49	**99.26**

## Data Availability

Not applicable.
